# Predicting pathologic response to neoadjuvant chemotherapy in patients with locally advanced breast cancer using multiparametric MRI

**DOI:** 10.1186/s12880-021-00688-z

**Published:** 2021-10-23

**Authors:** Nannan Lu, Jie Dong, Xin Fang, Lufang Wang, Wei Jia, Qiong Zhou, Lingyu Wang, Jie Wei, Yueyin Pan, Xinghua Han

**Affiliations:** 1 Department of Oncology, The First Affiliated Hospital of USTC, Division of Life Science and Medicine, University of Science and Technology of China, Lujiang Road 17, Hefei, 230001 Anhui China; 2 Department of Radiology, The First Affiliated Hospital of USTC, Division of Life Sciences and Medicine, University of Science and Technology of China, Hefei, 230031 Anhui China; 3 Department of Medical Oncology, Anhui Provincial Hospital Affiliated To Anhui Medical University, Hefei, 230032 Anhui China

**Keywords:** Breast cancer, Neoadjuvant chemotherapy, Standard apparent diffusion coefficient, Pathologic complete response

## Abstract

**Background:**

This study aims to observe and analyze the effect of diffusion weighted magnetic resonance imaging (MRI) on the patients with locally advanced breast cancer undergoing neoadjuvant chemotherapy.

**Methods:**

Fifty patients (mean age, 48.7 years) with stage II–III breast cancer who underwent neoadjuvant chemotherapy and preoperative MRI between 2016 and 2020 were retrospectively evaluated. The associations between preoperative breast MRI findings/clinicopathological features and outcomes of neoadjuvant chemotherapy were assessed.

**Results:**

Clinical stage at baseline (OR: 0.104, 95% confidence interval (CI) 0.021–0.516,* P* = 0.006) and standard apparent diffusion coefficient (ADC) change (OR: 9.865, 95% CI 1.024–95.021, *P* = 0.048) were significant predictive factors of the effects of neoadjuvant chemotherapy. The percentage increase of standard ADC value in pathologic complete response (pCR) group was larger than that in non-pCR group at first time point (*P* < 0.05). A correlation was observed between the change in standard ADC values and tumor diameter at first follow-up (r: 0.438, *P* < 0.05).

**Conclusions:**

Our findings support that change in standard ADC values and clinical stage at baseline can predict the effects of neoadjuvant chemotherapy for patients with breast cancer in early stage.

**Supplementary Information:**

The online version contains supplementary material available at 10.1186/s12880-021-00688-z.

## Background

The incidence and mortality of breast cancer rank first in women worldwide [1]. Neoadjuvant chemotherapy can reduce the tumor stage and postoperative recurrence rate, increase the resection rate and breast preservation rate, assess sensitivity to chemotherapeutic drugs in vivo, and guide clinical applications of postoperative adjuvant chemotherapy [2, 3]. As such, neoadjuvant chemotherapy plays an important role in the preoperative treatment of patients with locally advanced breast cancer. However, given the lack of predictive factors for neoadjuvant chemotherapy, it is not clear how to choose the chemotherapy regimen with the highest pathologic complete response rate.

Traditional clinical evaluation methods, such as breast X-ray and B-ultrasound, cannot accurately distinguish the nature of nodules or evaluate necrosis [4]. Due to its good soft tissue resolution and spatial resolution, magnetic resonance imaging (MRI) has numerous advantages over X-ray and B-ultrasound for detecting the extent and depth of tumors, simultaneous imaging comparison of double breast lesions, and monitoring recurrence after breast-conserving surgery [4]. Traditional MRI can be used to evaluate the effect of chemotherapy based on changes in tumor diameter and volume, which are often inconsistent with postoperative pathological results [5].

Diffusion weighted imaging (DWI) technology can measure and image the dispersion of water molecules by detecting the characteristics of dispersion motion [6]. Different from conventional MRI sequences, DWI can evaluate water molecule exchange in tissues and components under pathological and physiological conditions, which is expressed as the apparent diffusion coefficient (ADC). A high ADC value indicates fast molecular diffusion. Notably, the ADC values of malignant breast tumors are often lower than those of benign masses. Due to the small extracellular spaces and high cell density of tumor tissue, the movement of water molecules is limited in the malignant breast tumor microenvironment [7, 8].

Chemotherapy, as well as other toxic reactions, are commonly characterized by cell lysis and apoptosis. After chemotherapy or radiotherapy, the length, thickness, micro-vessel density, permeability, and blood flow velocity of blood vessels may change before the tumor size changes. Changes in cell membrane permeability caused by cell necrosis can lead to increased extracellular space and water molecular fluidity, which can significantly reduce the ADC value of tumors. Therefore, the ADC value may predict the efficacy of chemotherapy before imaging evaluation for tumor diameter.

Given its advantages for displaying and evaluating the blood supply, proliferation, vascular length, and cell density of small lesions, DWI is widely used for the diagnosis and differentiation of small nodules, evaluation of curative effects, and monitoring of recurrence [9–11]. Previous studies have reported that multiparametric MRI are closely related to the effects of chemotherapy, pathological grading, survival time, and positive margin after breast conserving surgery [8, 12–17].

In the present study, 50 patients with stage II–III breast cancer who received neoadjuvant chemotherapy were examined by MRI at different time points. Various MRI parameters were used in analyses of the relationships between the clinicopathological features of breast cancer patients and the effects of neoadjuvant chemotherapy and to determine the predictive value of MRI parameters.

## Methods

### Study population

**Fig. 1:**
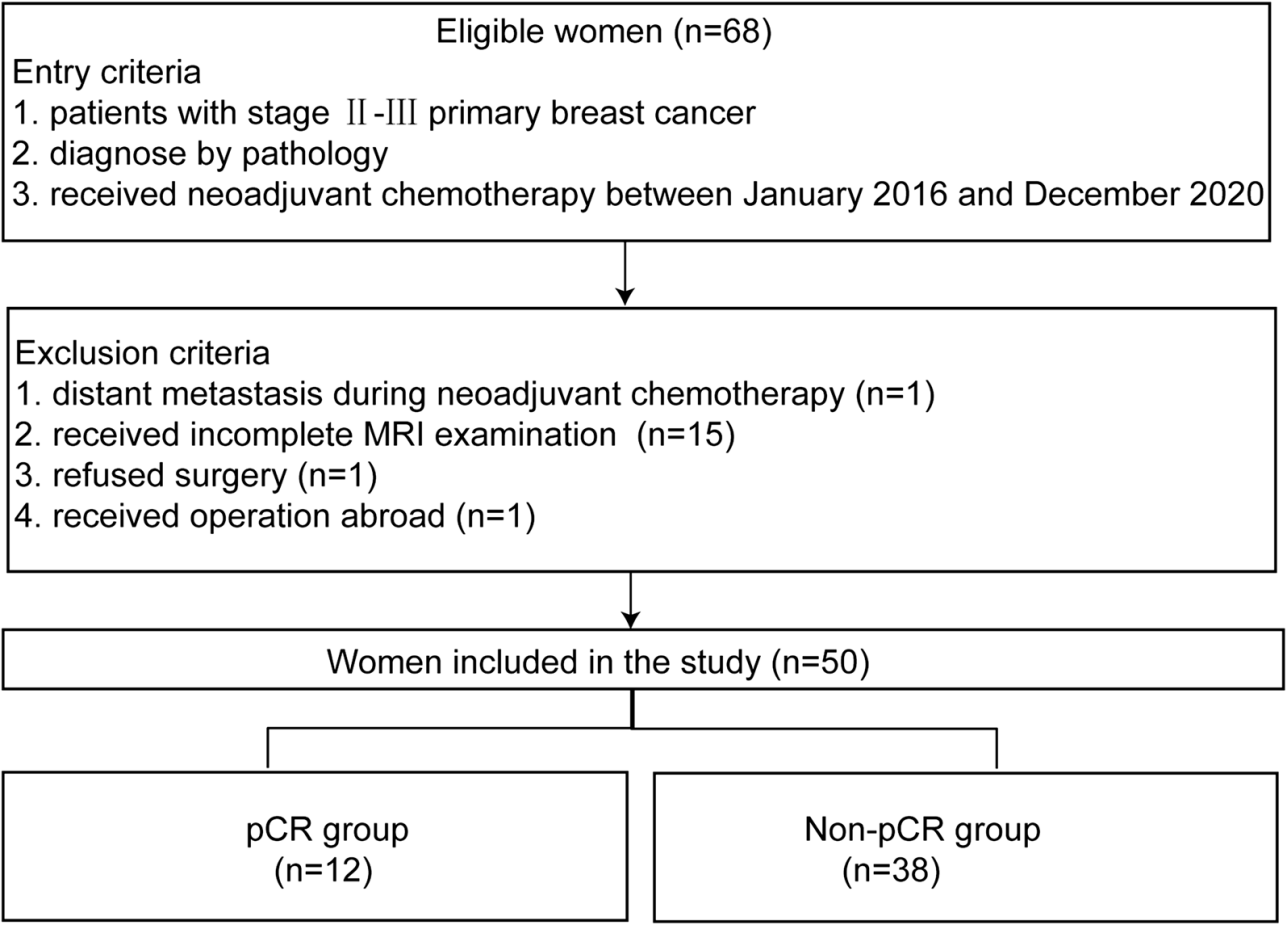
Flowchart of patient selection

Fifty patients with stage II–III breast cancer who underwent neoadjuvant chemotherapy and preoperative MRI between 2016 and 2020 were retrospectively included in this study. The inclusion and exclusion criteria are presented as a flowchart in Fig. 1. Patients with locally advanced breast cancer who met the inclusion/exclusion criteria were randomly treated with one of the following neoadjuvant chemotherapy regimens: EC (epirubicin plus cyclophosphamide), EC-TH (docetaxel plus herceptin), TEC (docetaxel, epirubicin, plus cyclophosphamide) and so on with two to eight cycles. If patients showed serious chemotherapy-related side effect, the dose was adjusted accordingly. In case of disease progression, neoadjuvant chemotherapy was discontinued.

### Pathological evaluation

Postoperative pathological sections were observed. According to postoperative pathological evaluation, the chemotherapeutic effect was graded as 1–5, according to the Miller and Payne classification criteria [18, 19]. Pathological reaction grade 5 was regarded as pathologic complete response (pCR), while pathological reaction grades 1–4 were regarded as not pathologic complete response (non-pCR).

### MRI examinations

All patients were imaged using a 3.0 T MRI (GE Signa HD × T, America) with an 8-channel dedicated breast coil. Conventional plain scan sequences included transverse T1WI, oblique sagittal T2WI fat suppression, and IVIM-DWI. Enhanced sequences included dynamic contrast enhanced MRI (DCE-MRI), delay-phase transgression, and vibrant sagittal enhancement. For the IVIM-DWI sequence, the parameters were: 9 b values of axial DWI; diffusion coefficient b values of 0, 25, 50, 100, 150,200, 500, 800, and 1000 s/mm^2^; FOV 38 cm × 26 cm; TR 4000 ms; TE 76.9 ms; layer thickness 4 mm; layer spacing 1.0 mm; and matrix 96 × 130. For the DCE-MRI sequence, the parameters were: FOV 38 × 32 cm, TR 4.2 ms, TE 2.0 ms, layer thickness 4 mm, layer spacing 0 mm, matrix 320 × 192, turning angle 15°, single phase temporal resolution 15 s, and a total of 40 phases. IVIM-DWI parameter values and color images were obtained through the GE ADW 4.5 workstation using FuncTool software. The DCE-MRI relevant parameters were calculated by omni-kinetics software (GE Healthcare, China). The region of interest (ROI) on the IVIM-DWI parameter map and DCE-MRI perfusion map was manually set by two senior radiologists with more than 10 years of experience in breast diagnosis. The ROIs for two sequences were set to remain consistent with reference to the T2WI fat suppression sequence, DWI sequence, and DCE-MR sequence images with arterial phase. The ROI of the tumor should cover at least 2/3 of the lesion, keeping away from cystic degeneration, bleeding, and the necrosis area as much as possible. Three apparent diffusion coefficients (ADC) were measured by a single exponential model, and the average value was obtained.

The analyzed MRI features in the study included breast type, lesion types, lesion location, lesion quadrant, BI-RADS rating, pectoral muscle invasive, skin around areola invasive, crater nipple, internal mammary artery thickening, subareolar duct invasive, contralateral breast, standard ADC change, tumor size change, slow ADC change, fast ADC, F value and TIC type, respectively.

### Statistical analysis

The Chi-squared test or Fisher’s exact test was used to assess the correlation between preoperative MRI findings or clinicopathological factors and MP grades. Multivariate analysis was performed using logistic regression, the odds ratios (ORs) and Person’s correlation coefficient (r) were estimated. We considered *P* values less than 0.05 to be statistically significant. SPSS software (version 25.0, IBM) was used for all statistical analyses.

## Results

### Relationship between clinicopathological features and chemotherapeutic effects

The final histopathological results of the surgical specimens revealed invasive carcinoma in 46 patients, invasive ductal carcinoma in 1 patients, ductal carcinoma in 1 patient, and mucinous adenocarcinoma in 2 patient (Table 1). Of the total 50 patients, 12 (24.0%) showed pCR and 38 (76.0%) showed non-pCR. The pCR and non- pCR groups did not differ significantly in terms of age (mean 45.0 ± 10.4 vs. 49.9 ± 10.6, respectively, *P* = 0.169, Table 1) or tumor size (mean 11.7 ± 11.7 vs. 11.3 ± 10.1, respectively, *P* = 0.936, Table 1). The relationships between clinicopathological features and chemotherapeutic effects were then studied. The clinical T stage was associated with chemotherapeutic effect (Table 1). Clinical stage (OR: 9.667, 95% CI 2.145–43.563, *P* = 0.003) and clinical T stage (OR: 0.119, 95% CI 0.027–0.530, *P* = 0.005) were significantly associated with chemotherapeutic effect in univariate regression analysis and were thus included in the multivariate logistic regression analysis (Table 2).

**Table 1:** Analysis of associations between clinicopathologic factors and effect of neoadjuvant chemotherapy

Variable	All, n (%)	pCR, n (%)	Non-pCR, n (%)	*P* value
Patient age (years)	48.7 ± 10.7	45.0 ± 10.4	49.9 ± 10.6	0.169
Tumor size (mm^3^)*	11.6 ± 11.2	11.7 ± 11.7	11.3 ± 10.1	0.936
Histological type*				
Invasive	47 (94.0)	11 (91.7)	36 (94.7)	
Other	3 (6.0)	1 (8.3)	2 (5.3)	0.696
Histologic grade*				
1–2	34 (68.0)	11 (91.7)	23 (60.5)	
3	16 (32.0)	1 (8.3)	15 (24.0)	0.060
Clinical stage*				
II	18 (36.0)	9 (75.0)	9 (76.0)	
III	32 (64.0)	3 (25.0)	29 (71.0)	0.001
T stage*				
T1–T2	19 (38.0)	9 (75.0)	10 (26.3)	
T3–T4	31 (62.0)	3 (25.0)	28 (73.7)	0.002
N stage*				
N0	4 (8.0)	1 (8.3)	3 (7.9)	
N1–3	46 (92.0)	11 (91.7)	35 (92.1)	0.961
Lymph node size*				
≤ 1.0 cm	29 (58.0)	7 (58.3)	22 (57.9)	
> 1.0 cm	21 (42.0)	5 (41.7)	16 (42.1)	0.979
NAC regimen				
EC	16 (32.0)	4 (33.3)	12 (31.6)	
EC-T(H)	24 (48.0)	6 (50.0)	18 (47.4)	
TEC or other	10 (20.0)	2 (16.7)	8 (21.1)	0.947
AC cycle				
1–4	16 (32.0)	3 (25.0)	13 (34.2)	
5–8	34 (68.0)	9 (75.0)	25 (65.8)	0.551
CEA level*				
Normal	45 (90.0)	11 (91.7)	34 (89.5)	
Abnormal	5 (10.0)	1 (8.3)	4 (10.5)	0.654
CA125 level*				
Normal	46 (92.0)	11 (91.7)	35 (92.1)	
Abnormal	4 (8.0)	1 (8.3)	3 (7.9)	0.961
CA153 level*				
Normal	45 (90.0)	10 (83.3)	35 (92.1)	
Abnormal	5 (10.0)	2 (16.7)	3 (7.9)	0.377
Breast cancer subtype				
TNBC	12 (24.0)	4 (33.3)	8 (21.1)	
Her-2 positive	10 (20.0)	2 (16.7)	8 (21.1)	
Luminal A(B)	28 (56.0)	6 (50.0)	22 (57.9)	0.683
Ki-67 status				
≤ 20%	15 (30.0)	2 (16.7)	13 (34.2)	
> 20%	35 (70.0)	10 (83.3)	25 (65.8)	0.248

*NAC* neoadjuvant chemotherapy, *TNBC* triple-negative breast cancer, *EC* epirubicin plus cyclophosphamide, *TH* docetaxel plus Herceptin, *TEC* docetaxel, epirubicin plus cyclophosphamide

^*^Data are measured at baseline. The data of patient age and tumor size are mean ± standard deviation

**Table 2:** Univariate logistic regression analysis of clinicopathologic factors and effect of neoadjuvant chemotherapy

Variable	Univariate analysis		
	OR	95% CI	*P* value
Patient age (years)			
< 50	0.450	0.116–1.751	0.249
≥ 50	Ref		
Histological type			
Invasive	1.636	0.135–19.808	0.699
Other	Ref		
Histologic grade*			
1–2	0.156	0.018–1.339	0.090
3	Ref		
Clinical stage*			
I–II	9.667	2.145–43.563	0.003
III	Ref		
T stage*			
T1–T2	0.119	0.027–0.530	0.005
T3–T4	Ref		
N stage*			
N0	0.943	0.089–10.010	0.961
N1–3	Ref		
Lymph node size*			
≤ 1.0 cm	0.982	0.263–3.662	0.979
> 2.0 cm	Ref		
NAC regimen			
EC	1.333	0.196–9.083	0.769
EC-T(H)	1.333	0.220–8.099	0.755
TEC or other	Ref		
NAC cycle			
1–4	1.560	0.359–6.775	0.553
5–8	Ref		
CEA level*			
Normal	0.600	0.063–5.709	0.657
Abnormal	Ref		
CA125 level*			
Normal	1.061	0.100–11.260	0.961
Abnormal	Ref		
CA153 level*			
Normal	2.333	0.341–15.952	0.388
Abnormal	Ref		
Breast cancer subtype			
TNBC	Ref		
Her-2 positive	0.500	0.070–3.550	0.488
Luminal A/B	0.545	0.121–2.449	0.429
Ki-67 status			
≤ 20%	2.600	0.495–13.668	0.259
> 20%	Ref		

*NAC* neoadjuvant chemotherapy, *TNBC* triple-negative breast cancer, *EC* epirubicin plus cyclophosphamide, *TH* docetaxel plus Herceptin, *TEC* docetaxel,epirubicin plus cyclophosphamide

*Data are measured at baseline. The data of patient age and tumor size are mean ± standard deviation

### Relationship between MRI parameters and chemotherapeutic effects

Tumor features and MRI parameters were analyzed using the chi-square test. The associations between MRI parameters and effects of neoadjuvant chemotherapy were present (Table 3). Table 4 summarizes the results of the univariate logistic regression analysis of MRI findings. Standard ADC value change (OR: 9.9, 95% CI 1.16–84.471, *P* = 0.036) and lesion location (OR: 0.217, 95% CI 0.051–0.936, *P* = 0.040) were strongly associated with the effects of neoadjuvant chemotherapy (Table 4). Lesion type (irregular) (OR: 0.182; 95% CI 0.027, 1.243; *P* = 0.082), lesion quadrant (outer quadrant) (OR: 0.182, 95% CI 0.027–1.243, *P* = 0.082), and tumor size change (OR: 0.156, 95% CI 0.018–1.339, *P* = 0.090) were weakly associated with the effects of neoadjuvant chemotherapy (Table 4). The factors with *P* < 0.1 were included in the multivariate logistic regression and the included factors were lesion types, lesion location, lesion quadrant, standard ADC change and tumor size change, respectively (Table 5). The multivariate logistic regression analysis showed that clinical stage (OR: 0.104, 95% CI 0.021–0.516, *P* = 0.006) and standard ADC value change (OR: 9.865, 95% CI 1.024–95.021, *P* = 0.048) were predictive factors of the effects of neoadjuvant chemotherapy (Table 5).

**Table 3:** Analysis of associations between MRI findings and effect of neoadjuvant chemotherapy

Variable	All, n (%)	pCR, n (%)	Non-pCR, n (%)	*P* value
Breast type				
Fibrous gland	14 (28.0)	3 (25.0)	9 (23.7)	
Compact	30 (60.0)	7 (58.3)	5 (13.2)	
Unknown	6	2	4	0.936
Lesion types				
Irregular	26 (52.0)	4 (33.3)	22 (57.9)	
Section distribution	6 (12.0)	3 (25.0)	3 (7.9)	
Circular	8 (16.0)	3 (25.0)	5 (13.2)	
Satellite lesions	10 (20.0)	2 (16.7)	8 (21.1)	0.244
Lesion location				
Left	24 (48.0)	9 (75.0)	15 (39.5)	
Right	26 (52.0)	3 (25.0)	23 (60.5)	0.032
Lesion quadrant				
Outer quadrant	37 (74.0)	9 (75.0)	28 (73.7)	
Upper inner	7 (14.0)	1 (8.3)	6 (15.8)	
Central	5 (10.0)	1 (8.3)	4 (10.5)	
Diffuse	5 (10.0)	1 (8.3)	4 (10.5)	0.886
BI-RADS rating				
5	39 (78.0)	9 (75.0)	30 (78.9)	
6	8 (16.0)	2 (16.7)	6 (15.8)	
Unknown	3	1	2	0.573
Pectoral muscle invasive				
Yes	13 (26.0)	10 (83.3)	3 (7.9)	
No	37 (74.0)	2 (16.7)	35 (92.1)	0.398
Skin around areola invasive				
Yes	30 (60.0)	5 (41.7)	25 (65.8)	
No	20 (40.0)	7 (58.3)	13 (34.2)	0.137
Crater nipple				
Yes	18 (36.0)	2 (16.7)	16 (42.1)	
No	32 (64.0)	10 (83.3)	22 (57.9)	0.109
Internal mammary artery thickening				
Yes	30 (60.0)	7 (58.3)	23 (60.5)	
No	20 (40.0)	5 (41.7)	15 (39.5)	0.892
Subareolar duct invasive				
Yes	18 (36.0)	3 (25.0)	15 (39.5)	
No	32 (64.0)	9 (75.0)	24 (63.2)	0.109
Contralateral breast				
Hyperplasia and adenosis	30 (60.0)	9 (75.0)	21 (55.3)	
Galactocele/fortified nodule	14 (28.0)	1 (8.3)	12 (31.6)	
No	8 (16.0)	2 (16.7)	6 (15.8)	0.202
Standard ADC change*				
≤ 15%	19 (38.0)	1 (18.3)	18 (47.4)	
> 15%	31 (62.0)	11 (91.7)	20 (52.6)	0.015
Slow ADC change*				
≤ 15%	17 (36.2)	2 (16.7)	15 (42.9)	
> 15%	30 (63.8)	10 (83.3)	20 (57.1)	0.103
Unknown	3	1	2	
Tumor size change*				
≤ 15%	15 (30.0)	1 (8.3)	14 (36.8)	0.06
> 15%	35 (70.0)	11 (91.7)	24 (63.2)	0
Fast ADC (10^−3 ^mm^2^/s)				
Value	15.0 ± 14.4	13.9 ± 17.9	15.3 ± 13.1	0.785
Unknown	6	1	5	
F value (%)				
Value	40.3 ± 27.8	47.3 ± 30.7	38.0 ± 26.9	
Unknown	5	1	4	0.337
TIC type				
Ascending	6 (12.0)	1 (8.3)	5 (13.2)	
Reduced	15 (30.0)	5 (41.7)	10 (26.3)	
Invariable	29 (58.0)	6 (50.0)	23 (60.5)	0.586

MRI data are measured at baseline

*ADC* apparent diffusion coefficient, *TIC* time intensity curve, *F value* fraction of fast ADC

*Data are measured at the first two cycles

**Table 4:** Univariate logistic regression analysis of MRI findings and effect of neoadjuvant chemotherapy

Variable	Univariate analysis		
	OR	95% CI	*P* value
Breast type			
Fibrous gland	0.682	0.085–5.448	0.718
Compact	0.795	0.125–5.045	0.808
Unknown	Ref		
Lesion types			
Irregular	0.182	0.027–1.243	0.082
Section distribution	Ref		
Circular	0.600	0.070–5.136	0.641
Satellite lesions	0.250	0.027–2.319	0.223
Lesion location			
Left	0.217	0.051–0.936	0.040
Right	Ref		
Lesion quadrant			
Outer quadrant	0.182	0.027–1.243	0.082
Upper inner	0.600	0.070–5.136	0.641
Central	Ref		
Diffuse	0.250	0.027–2.319	0.223
BI-RADS rating			
5	1.667	0.306–9.080	0.555
6	Ref		
Unknown	5.000	0.212–117.894	0.318
Pectoral muscle invasive			
Yes	2.037	0.383–10.845	0.404
No	Ref		
Skin around areola invasive			
Yes	2.692	0.713–10.170	0.144
No	Ref		
Crater nipple			
Yes	3.636	0.699–18.918	0.125
No	Ref		
Internal mammary artery thickening			
Yes	1.095	0.293–4.097	0.892
No	Ref		
Subareolar duct invasive			
Yes	3.636	0.699–18.918	0.125
No	Ref		
Contralateral breast			
Hyperplasia and adenosis	6.158	0.694–54.644	0.103
Galactocele/fortified nodule	Ref		
No	4.333	0.326–57.649	0.267
Standard ADC change*			
≤ 15%	9.9	1.16–84.471	0.036
> 15%	Ref		
Tumor size change*			
≤ 15%	0.156	0.018–1.339	0.090
> 15%	Ref		
Slow ADC change*			
≤ 15%	3.75	0.714–19.707	0.118
> 15%	Ref		
TIC ype			
Ascending	0.400	0.036–4.411	0.454
Reduced	Ref		
Invariable	0.522	0.129–2.116	0.362

MRI data are measured at baseline

*ADC* apparent diffusion coefficient, *TIC* time intensity curve

*Data are measured at the first two cycles

**Table 5:** Multivariate logistic regression analysis of MRI findings and effect of neoadjuvant chemotherapy

Variable	Multivariate analysis		
	OR	95% CI	*P* value
Clinical stage*			
I–II	0.104	0.021–0.516	0.006
III	Ref		
Standard ADC change*			
≤ 15%	9.865	1.024–95.021	0.048
> 15%	Ref		

MRI data are measured at baseline

*ADC* apparent diffusion coefficient

*Data are measured at the first two cycles

### Changes in standard ADC values between pCR and non-pCR group patients

**Fig. 2:**
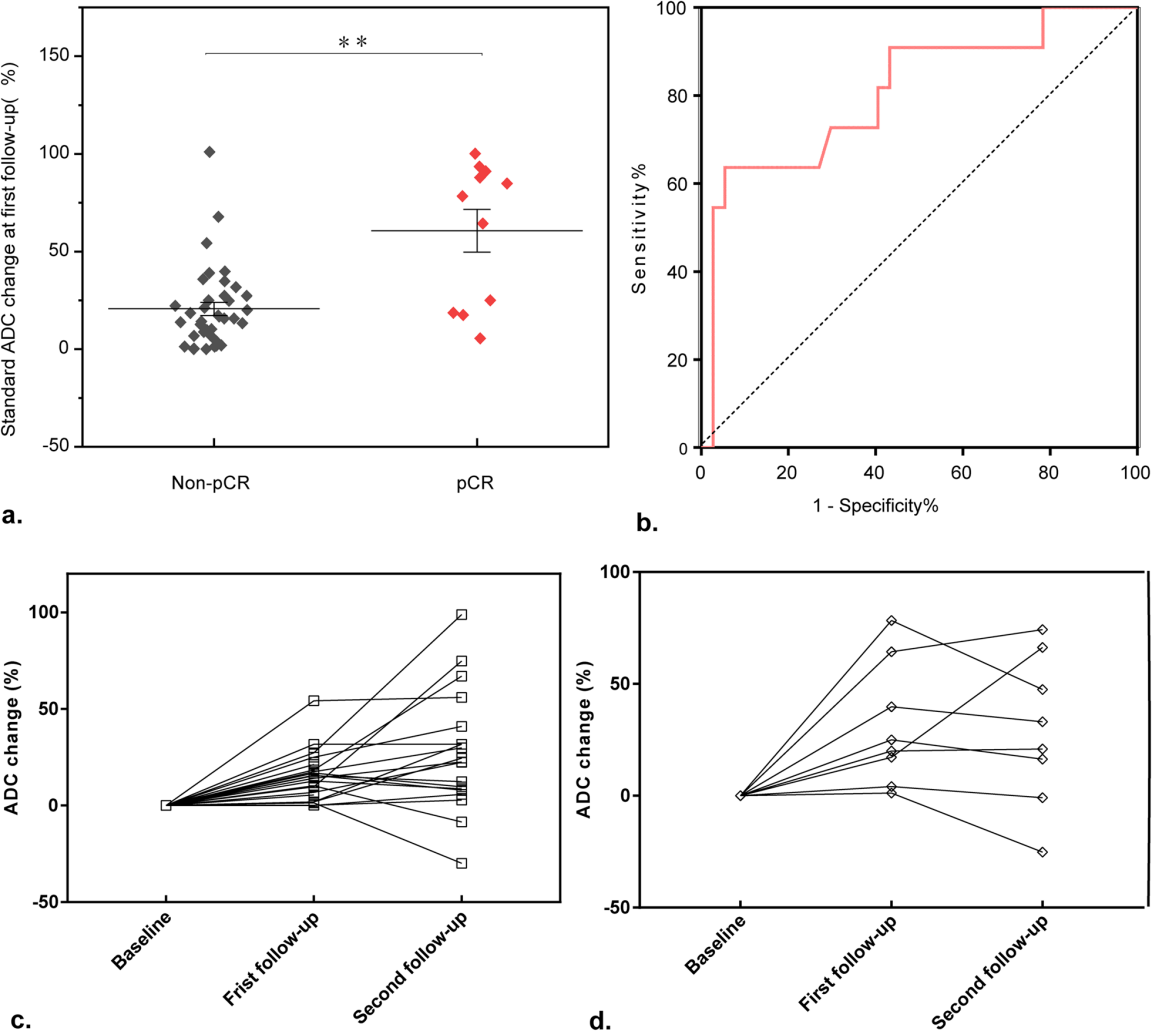
Plots of standard ADC changes in the non-pCR and pCR groups. **a** Changes in ADC values in non-pCR and pCR groups. Statistical significance was assessed at *P* < 0.05. **b** ROC curve. The AUC of the ROC curve was 0.828, 95% CI was 0.681–0.975, and the *P* value < 0.01. Changing trend in standard ADC values at different points in non-pCR (**c**) and pCR (**d**) groups

Changes in standard ADC values differed significantly between patients in the pCR and non-pCR group at first follow-up (*P* < 0.05, Fig. 2a). A plot of the receiver operating characteristic (ROC) curve is shown in Fig. 2b (Area under the ROC curve (AUC): 0.828, 95% CI 0.681–0.975, *P* < 0.05). Figure 2c shows the changes in standard ADC values in the pCR and non-pCR groups at baseline, first follow-up point, and second follow-up point. Percentage increase of the standard ADC value in the pCR group was larger than that in the non-pCR group at the first time point (*P* < 0.05, Fig. 2c, d). The changes in standard ADC values did not differ significantly between the two groups at the second time point (Fig. 2c, d). These findings suggest that changes in standard ADC values can predict the effects of neoadjuvant chemotherapy in the early stages.

### Correlation between standard ADC value and tumor diameter at different observation points

**Fig. 3:**
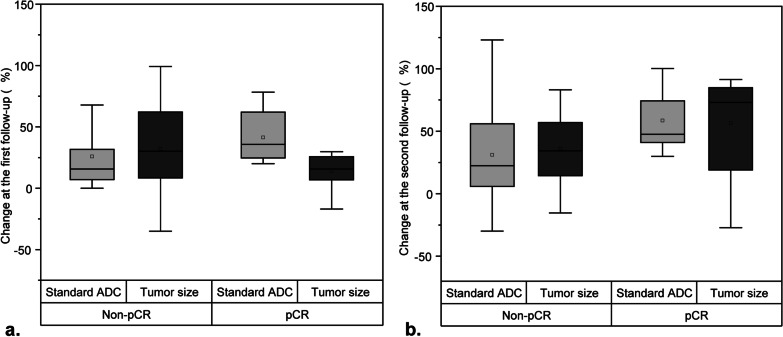
Box plot showed the change between standard ADC value and tumor size at observation points (**a**, **b**) in pCR versus non-pCR groups

**Fig. 4:**
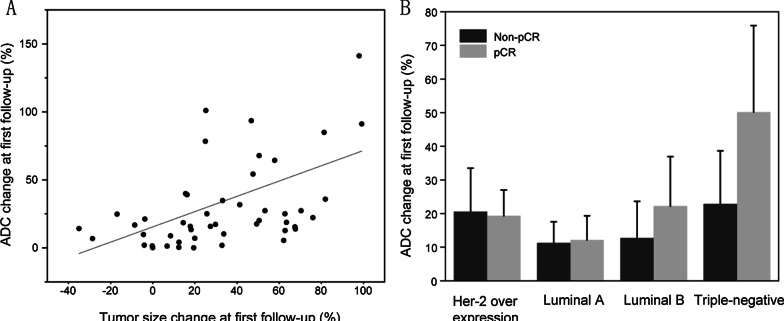
Plots of standard ADC changes at first follow-up and for the different breast cancer subtypes. **a** Correlation between change in the standard ADC value and change in tumor diameter. Pearson's r was 0.438 and *P* < 0.01. **b** Standard ADC values of different breast cancer subtypes in non-pCR and pCR groups

The correlation between tumor diameter and standard ADC value change for patients in both groups was assessed at the different time point (Fig. 3a, b). The percentage change of the standard ADC value in the pCR group was significantly higher than the change in tumor size at first follow-up, whereas there was no significant difference between percentage change in standard ADC value and change in tumor size in the non-pCR group at first follow-up (Fig. 3a, b). There was a significant correlation between change in the standard ADC value and tumor diameter at first follow-up (r = 0.438, *P* < 0.01; Fig. 4a). These results showed that changes in standard ADC values appeared at early stages and the values in the pCR group can predict the effects of neoadjuvant chemotherapy in breast cancer patients in the early stages.

### Comparison of standard ADC values between breast cancer subtypes

Changes in the standard ADC values for breast cancer patients with different molecular subtypes in the pCR and non-pCR groups were investigated at the first observation point. Patients with triple-negative type in pCR group had a slightly higher change in standard ADC value than that in non-pCR group at first follow-up (*P* > 0.01, Fig. 4b). At first follow-up, there were no significant differences in the percentage change of the standard ADC values among the other breast cancer subtypes (Fig. 4b).

### MRI findings and ADC maps of two patients at different time points

**Fig. 5:**
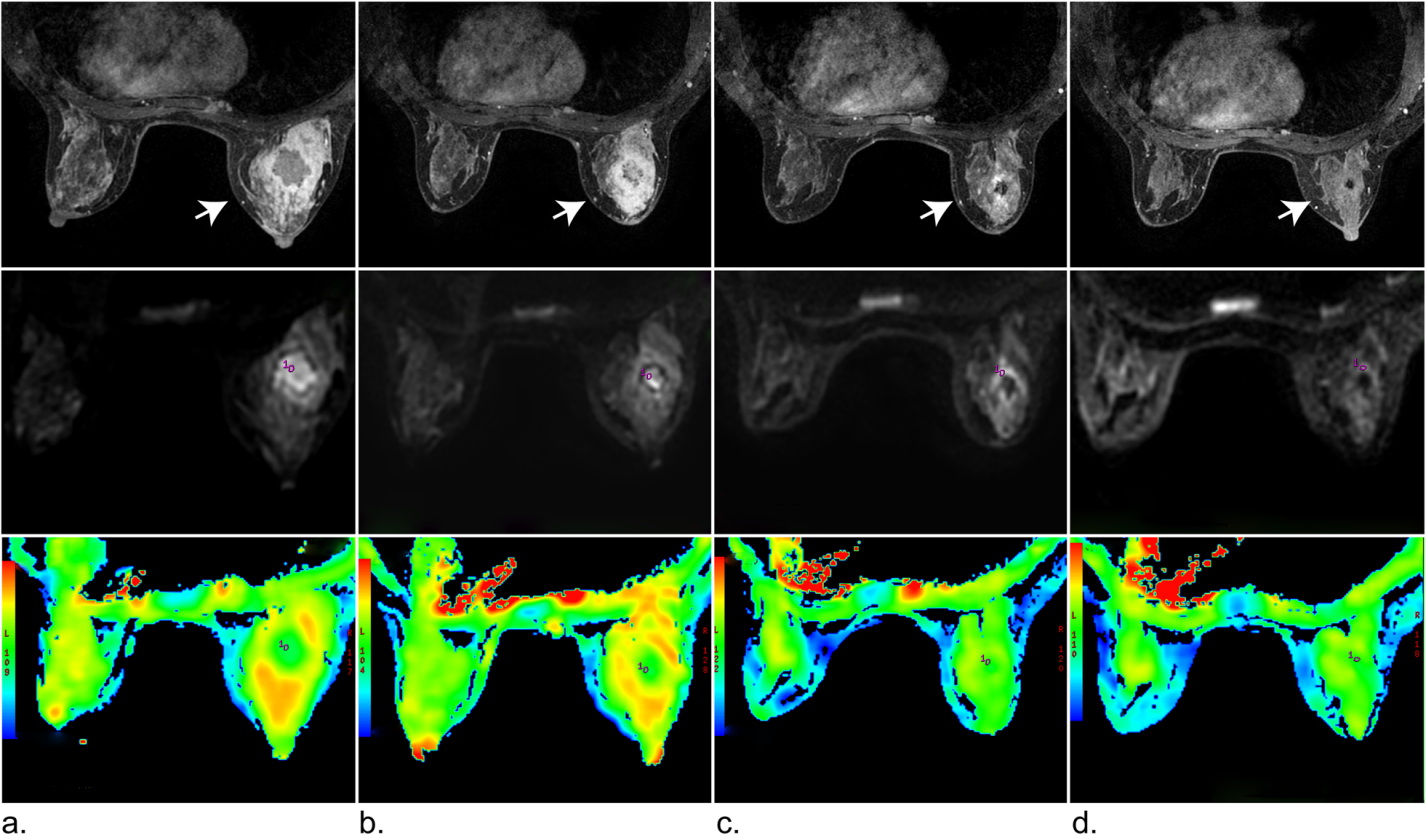
MRI images of a 49-year-old woman with invasive breast cancer who showed pCR after completing neoadjuvant chemotherapy (six cycles EC + T). **a**–**d** From top to bottom, the images show the vibrant enhancement sequence, IVIMnd ADC map, respectively. The ROI area of the DWI images is marked by a red circle. At baseline, first follow-up, second follow-up, and preoperatively, the tumor volume (white arrow) was 29.93, 22.44, 2.60, and 0.4 cm^2^, respectively, and standard ADC value was 1.0 × 10^−3^, 1.3 × 10^−3^, 1.5 × 10^−3^ and 1.3 × 10^−3^, respectively

**Fig. 6:**
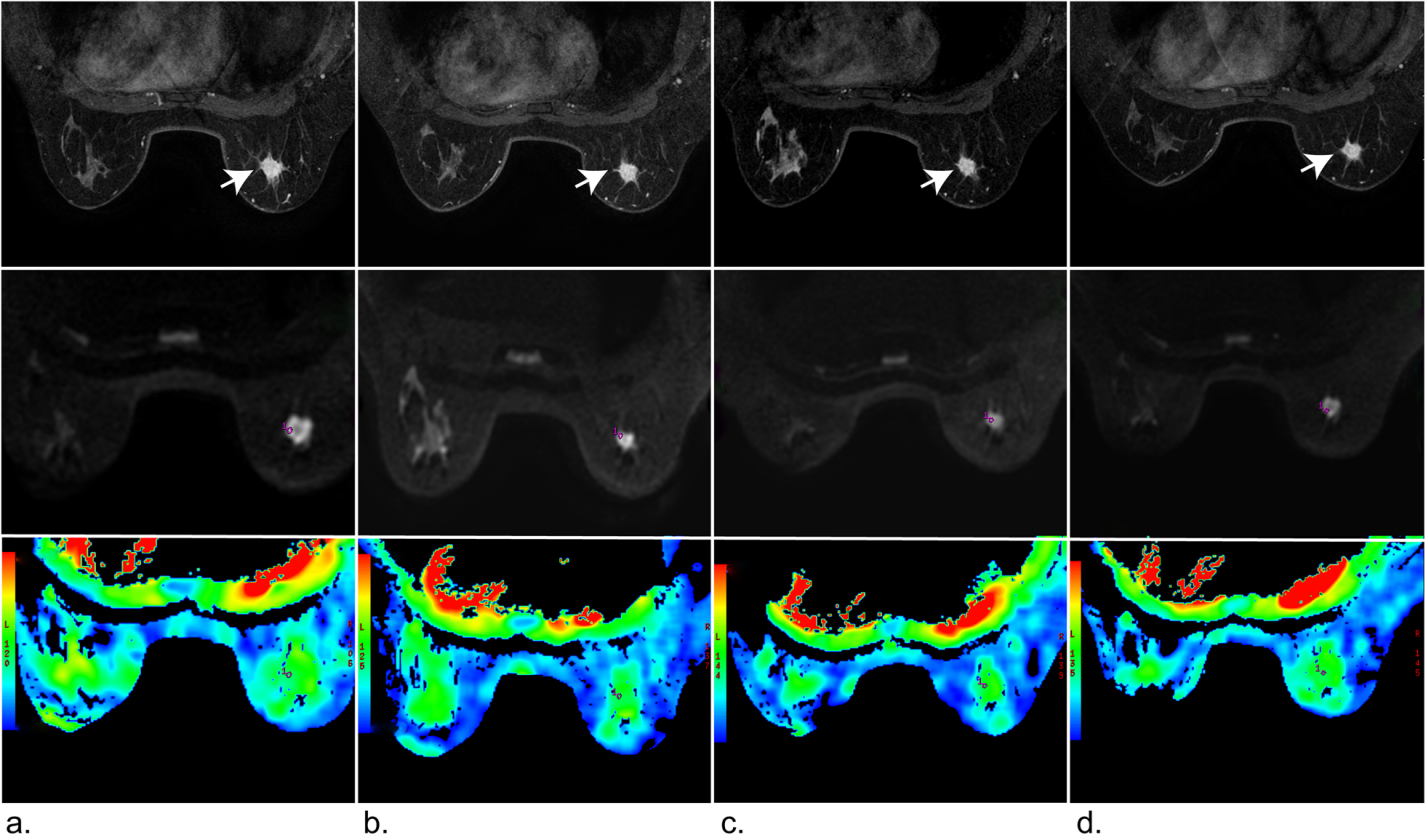
MRI images of a 51-year-old woman with invasive breast cancer who showed non-pCR after completing neoadjuvant chemotherapy (four cycles EC-T). **a**–**d** From top to bottom, the images show the vibrant enhancement sequence, IVIM DWI, and ADC map, respectively. The ROI area of the DWI images is marked by a red circle. At baseline, first follow-up, second follow-up, and preoperatively, the tumor volume (white arrow) was 2.40, 2.10, 2.08, and 2.09 cm^2^, respectively, and the standard ADC value was 1.21 × 10^−3^, 1.18 × 10^−3^, 1.20 × 10^−3^, and 1.22 × 10^−3^ mm^2^/s, respectively

A 49-year-old woman received six cycles of neoadjuvant chemotherapy (EC-T) and showed pCR (Fig. 5). At baseline, first follow-up, second follow-up, and preoperatively, the tumor volume decreased significantly and the standard ADC value increased gradually (Fig. 5). A 51-year-old woman received four cycles of neoadjuvant chemotherapy (EC-T) and showed non-pCR (Fig. 6). At baseline, first follow-up, second follow-up, and preoperatively, the tumor volume showed no obvious changes (2.40 cm^2^, 2.10 cm^2^, 2.08 cm^2^, 2.09 cm^2^, respectively) and the standard ADC values were also stable (1.21 × 10^−3^, 1.18 × 10^−3^, 1.20 × 10^−3^, and 1.22 × 10^−3^ mm^2^/s, respectively) (Fig. 6).

### MRI findings and TIC curves of two patients at different time points

The 3DFSPGR dynamic enhancement sequence and TIC curve at baseline, first follow-up, second follow-up, and preoperatively are shown for patients in Additional file 1: Fig. S1. The patient who showed pCR had TIC curve types with efflux-influx-influx-influx (Additional file 1: Fig. S1A–B). The patient who showed non-pCR had TIC curve types with efflux–efflux–efflux–influx (Additional file 1: Fig. S1C–D).

Sometimes the images to be treated are affected by uncertainties and / or inaccuracies such as to require a fuzzy preprocessing of the same [20, 21].

## Discussion

Chemotherapy can induce apoptosis and necrosis of tumors, resulting in a decreased density of tumor cells, incomplete tumor cell membranes, and increased extracellular space. The ADC value of a tumor is known to change when patients receive effective chemotherapy in the early stage of tumor progression [22–24]. Previous studies have also proposed that ADC values reflect ex vivo cell density and are correlated with apoptosis. Thus, ADC values may be a responsive marker for chemotherapeutic efficacy [25, 26].

Li et al. suggested a correlation between ADC value and the effect of neoadjuvant chemotherapy for breast cancer [12]. After the first cycle of neoadjuvant chemotherapy, the ADC value of tumor tissue increased significantly in patients who received complete response or partial response. Changes in the ADC values of tumor tissue after chemotherapy were positively correlated with changes in tumor diameter, and early changes in ADC values predicted the chemotherapy sensitivity of tumor tissue [12].

Our study found that, after the first cycle of neoadjuvant chemotherapy, the percentage increase of the standard ADC value in breast cancer patients in the pCR group was significantly higher than that in breast cancer patients in the non-pCR group (*P* < 0.01). Furthermore, a significant correlation between standard ADC value and tumor diameter was observed at first follow-up (r = 0.438; *P* < 0.01), which is similar to the results of previous studies. We found no significant difference in standard ADC value or tumor diameter between the pCR and non-pCR groups at second follow-up.

A retrospective study of 53 patients with locally advanced breast cancer by Sang et al. suggested that DW-MRI imaging can predict the effects of chemotherapy and guide clinical applications [27]. They reported that the ADC values of 36 patients who responded to treatment were significantly lower than those of patients who did not respond to treatment. Furthermore, the percentage change of the ADC value in patients who responded to treatment was significantly higher than that of patients who did not respond to treatment. These results indicate that patients with a lower ADC value before neoadjuvant therapy may benefit more from chemotherapy [27]. In our study, we also analyzed differences in standard ADC values at baseline for patients in different therapeutic groups. The standard ADC value of the 12 pCR patients (0.90 ± 0.18) before treatment was lower than that of the 38 non-pCR patients (0.99 ± 0.27) before treatment, although the difference was not statistically significant (*P* = 0.312).

Previous studies have observed a correlation between the ADC value and chemotherapy effect in breast cancer patients with different subtypes. One study reported that the ADC values in patients with triple-negative and Her-2 overexpression types were significantly higher than those in patients with luminal A and luminal B types before chemotherapy [10]. Furthermore, the ADC values in patients with triple-negative cancer were significantly higher than those in patients with other subtypes after chemotherapy, and the ADC values of patients with the triple-negative type in the pCR group before chemotherapy were significantly lower than those of patients in the non-pCR group [10]. Enida et al. found that ADC value was a predictive marker in some breast cancer subtypes [28]. Their study found a significant difference in ADC values between responsive patients and non-responsive patients with triple-negative and Her-2 overexpression type cancer [28]. In our study, we also observed a relationship between standard ADC values and chemotherapeutic efficacy in patients with different breast cancer subtypes. We found that there was no difference in standard ADC values in all breast cancer subtypes before chemotherapy. However, the standard ADC values in patients with triple-negative cancer at first follow-up differed between the pCR and non-pCR groups. The standard ADC values in patients with triple-negative cancer in the pCR group were significantly higher than that in the non-pCR group, although the difference was not statistically significant (*P* > 0.05).

Lastly, previous studies have reported that the TIC curve of breast lesions can reflect the micro-vessel density of tissue and vascular permeability, which are valuable for the diagnosis of benign and malignant breast lesions [29, 30]. In recent years, studies have found that changes in the TIC curve type can correlate with the prognosis of neoadjuvant chemotherapy in breast cancer patients [31, 32]. However, in our analysis of 50 patients before and after neoadjuvant chemotherapy, we found no difference in the TIC curve type between the pCR and non-pCR groups. Only 5 of 12 pCR patients showed efflux-influx-influx type.

## Conclusions

This study aimed to investigate the clinicopathological features and MRI parameters of 50 breast cancer patients who received neoadjuvant chemotherapy. Our analysis showed that the clinical stage at baseline and changes in standard ADC values were closely related to the effects of neoadjuvant chemotherapy. The standard ADC values may change before any reduction in tumor size, thus predicting the neoadjuvant effects before imaging evaluation.

## Supplementary Information

**Additional file 1: Figure S1.** Different MRI sequences for patients in the pCR and non-pCR groups. From top to bottom, the images show the 3DFSPGR dynamic enhancement sequence and TIC curve, respectively, at baseline, first follow-up, second follow-up, and preoperatively. The tumor is marked by a white arrow and the ROI area of the MRI is marked by a red circle. (A–B) MRI images of a 58-year-old woman with invasive breast cancer who showed pCR after completing neoadjuvant chemotherapy (eight cycles AC-TH). From top to bottom, the TIC type was efflux, influx, influx, and influx, respectively. (C–D) MRI images of a 45-year-old woman with invasive breast cancer who showed non-pCR after completing neoadjuvant chemotherapy (eight cycles EC-TH). From top to bottom, the TIC type was efflux, efflux, efflux, and influx, respectively.

## Data Availability

The datasets analyzed in this study are available from the corresponding author on request.

## Abbreviations

pCR: Pathologic complete response
CI: Confidence interval
ADC: Apparent diffusion coefficient
ROC: Receiver operating characteristic
AUC: Area under the ROC curve
DCE: Dynamic contrast enhanced
DWI: Diffusion-weighted imaging

## References

[1] Siegel RL, Miller KD, Jemal A (2015). Cancer statistics, 2015. CA Cancer J Clin.

[2] Killelea BK, Yang VQ, Mougalian S, Horowitz NR, Pusztai L, Chagpar AB (2015). Neoadjuvant chemotherapy for breast cancer increases the rate of breast conservation: results from the national cancer database. J Am Coll Surg.

[3] Gralow JR, Burstein HJ, Wood W, Hortobagyi GN, Gianni L, von Minckwitz G (2008). Preoperative therapy in invasive breast cancer: pathologic assessment and systemic therapy issues in operable disease. J Clin Oncol.

[4] McLaughlin R, Hylton N (2011). MRI in breast cancer therapy monitoring. NMR Biomed.

[5] Kwong MS, Chung GG, Horvath LJ, Ward BA, Hsu AD, Carter D (2006). Postchemotherapy MRI overestimates residual disease compared with histopathology in responders to neoadjuvant therapy for locally advanced breast cancer. Cancer J.

[6] Wang Q, Guo Y, Zhang J, Wang Z, Huang M, Zhang Y (2016). Contribution of IVIM to conventional dynamic contrast-enhanced and diffusion-weighted MRI in differentiating benign from malignant breast masses. Breast Care.

[7] Shao G, Fan L, Zhang J, Dai G, Xie T (2017). Association of DW/DCE-MRI features with prognostic factors in breast cancer. Int J Biol Mark.

[8] Partridge SC, Nissan N, Rahbar H, Kitsch AE, Sigmund EE (2017). Diffusion-weighted breast MRI: clinical applications and emerging techniques. J Magn Reson Imaging.

[9] Yuan J, Wong OL, Lo GG, Chan HHL, Wong TT, Cheung PSY (2016). Statistical assessment of bi-exponential diffusion weighted imaging signal characteristics induced by intravoxel incoherent motion in malignant breast tumors. Quant Imaging Med Surg.

[10] Liu S, Ren R, Chen Z, Wang Y, Fan T, Li C (2015). Diffusion-weighted imaging in assessing pathological response of tumor in breast cancer subtype to neoadjuvant chemotherapy. J Magn Reson Imaging.

[11] Galban CJ, Ma B, Malyarenko D, Pickles MD, Heist K, Henry NL (2015). Multi-site clinical evaluation of DW-MRI as a treatment response metric for breast cancer patients undergoing neoadjuvant chemotherapy. PLoS ONE.

[12] Li XR, Cheng LQ, Liu M, Zhang YJ, Wang JD, Zhang AL (2012). DW-MRI ADC values can predict treatment response in patients with locally advanced breast cancer undergoing neoadjuvant chemotherapy. Med Oncol.

[13] Baek H-M, Chen J-H, Nie K, Yu HJ, Bahri S, Mehta RS (2009). Predicting pathologic response to neoadjuvant chemotherapy in breast cancer by using MR imaging and quantitative1h mr spectroscopy. Radiology.

[14] Loo CE, Teertstra HJ, Rodenhuis S, van de Vijver MJ, Hannemann J, Muller SH (2008). Dynamic contrast-enhanced MRI for prediction of breast cancer response to neoadjuvant chemotherapy: initial results. Am J Roentgenol.

[15] Chen B-B, Lu Y-S, Yu C-W, Lin C-H, Chen TW-W, Wei S-Y (2018). Imaging biomarkers from multiparametric magnetic resonance imaging are associated with survival outcomes in patients with brain metastases from breast cancer. Eur Radiol.

[16] Uematsu T, Kasami M, Yuen S (2010). Neoadjuvant chemotherapy for breast cancer: correlation between the baseline MR imaging findings and responses to therapy. Eur Radiol.

[17] Kang JH, Youk JH, Kim JA, Gweon HM, Eun NL, Ko KH (2018). Identification of preoperative magnetic resonance imaging features associated with positive resection margins in breast cancer: a retrospective study. Korean J Radiol.

[18] Choi M, Park YH, Ahn JS, Im YH, Nam SJ, Cho SY (2016). Assessment of pathologic response and long-term outcome in locally advanced breast cancers after neoadjuvant chemotherapy: comparison of pathologic classification systems. Breast Cancer Res Treat.

[19] Romero A, Garcia-Saenz JA, Fuentes-Ferrer M, Garcia-Asenjo JAL, Furio V, Roman JM (2013). Correlation between response to neoadjuvant chemotherapy and survival in locally advanced breast cancer patients. Ann Oncol.

[20] Versaci M, Calcagno S, Morabito FC. Image contrast enhancement by distances among points in fuzzy hyper-cubes. In: 16th International conference on computer analysis of images and patterns (CAIP): Sep 02–04 2015; Valletta, MALTA. Berlin: Springer; 2015. pp. 494–505.

[21] Versaci M, Calcagno S, Morabito FC. IEEE: fuzzy geometrical approach based on unit hyper-cubes for image contrast enhancement. In: IEEE 2015 international conference on signal and image processing applications (ICSIPA): Oct 19–21 2015. Kuala Lumpur, Malaysia: IEEE; 2015. pp. 488–493.

[22] Patterson DM, Padhani AR, Collins DJ (2008). Technology Insight: water diffusion MRI—a potential new biomarker of response to cancer therapy. Nat Clin Pract Oncol.

[23] Galban CJ, Hoff BA, Chenevert TL, Ross BD (2017). Diffusion MRI in early cancer therapeutic response assessment. NMR Biomed.

[24] Afaq A, Andreou A, Koh DM (2010). Diffusion-weighted magnetic resonance imaging for tumour response assessment: Why, when and how?. Cancer Imaging.

[25] Fliedner FP, Engel TB, El-Ali HH, Hansen AE, Kjaer A (2020). Diffusion weighted magnetic resonance imaging (DW-MRI) as a non-invasive, tissue cellularity marker to monitor cancer treatment response. BMC Cancer.

[26] Zhang X-Y, Sun Y-S, Tang L, Xue W-C, Zhang X-P (2011). Correlation of diffusion-weighted imaging data with apoptotic and proliferation indexes in CT26 colorectal tumor homografts in balb/c mouse. J Magn Reson Imaging.

[27] Park SH, Moon WK, Cho N, Song IC, Chang JM, Park I-A (2010). Diffusion-weighted MR Imaging: pretreatment prediction of response to neoadjuvant chemotherapy in patients with breast cancer. Radiology.

[28] Bufi E, Belli P, Costantini M, Cipriani A, Di Matteo M, Bonatesta A (2015). Role of the apparent diffusion coefficient in the prediction of response to neoadjuvant chemotherapy in patients with locally advanced breast cancer. Clin Breast Cancer.

[29] Yin J, Yang J, Jiang Z (2018). Discrimination between malignant and benign mass-like lesions from breast dynamic contrast enhanced MRI: semi-automatic vs. manual analysis of the signal time-intensity curves. J Cancer.

[30] Kuhl CK, Mielcareck P, Klaschik S, Leutner C, Wardelmann E, Gieseke J (1999). Dynamic breast MR imaging: Are signal intensity time course data useful for differential diagnosis of enhancing lesions?. Radiology.

[31] Yuan C, Jin F, Guo X, Zhao S, Li W, Guo H (2019). Correlation analysis of breast cancer DWI combined with DCE-MRI imaging features with molecular subtypes and prognostic factors. J Med Syst.

[32] Fan X, Wu M, Lu L, Zhang Q, Guo Q, Zhao F (2016). Relating doses of contrast agent administered to TIC and semi-quantitative parameters on DCE-MRI: based on a murine breast tumor model. PLoS ONE.

