# Supplementary figures and images for: Predicting pathologic response to neoadjuvant chemotherapy in patients with locally advanced breast cancer using multiparametric MRI
Source: BMC Med Imaging. 2021 Oct 23;21:155. doi: 10.1186/s12880-021-00688-z (PMC8542288; doi:10.1186/s12880-021-00688-z)

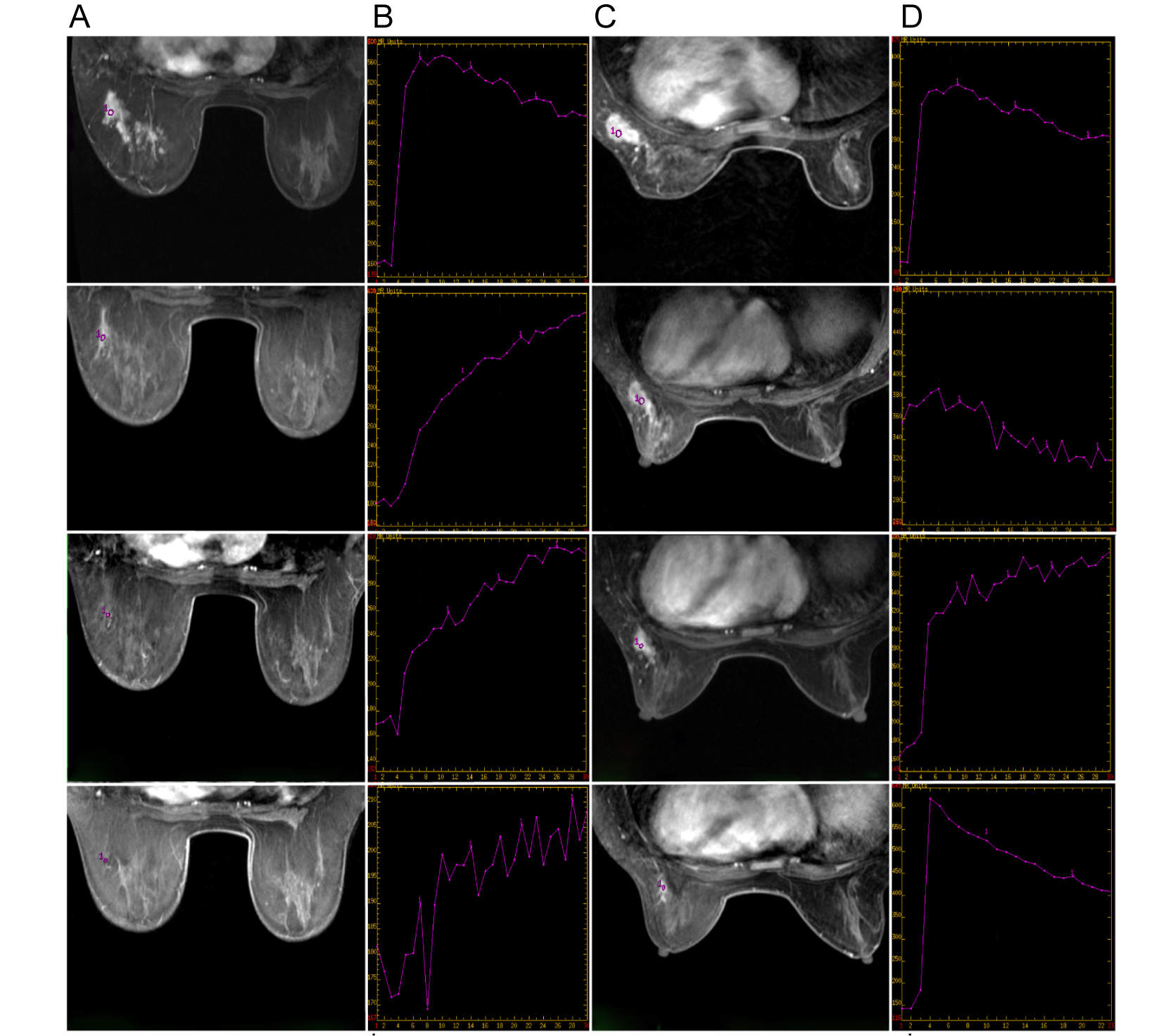

Supplement: Supplementary file 1 — Additional file 1: Figure S1. Different MRI sequences for patients in the pCR and non-pCR groups. From top to bottom, the images show the 3DFSPGR dynamic enhancement sequence and TIC curve, respectively, at baseline, first follow-up, second follow-up, and preoperatively. The tumor is marked by a white arrow and the ROI area of the MRI is marked by a red circle. (A–B) MRI images of a 58-year-old woman with invasive breast cancer who showed pCR after completing neoadjuvant chemotherapy (eight cycles AC-TH). From top to bottom, the TIC type was efflux, influx, influx, and influx, respectively. (C–D) MRI images of a 45-year-old woman with invasive breast cancer who showed non-pCR after completing neoadjuvant chemotherapy (eight cycles EC-TH). From top to bottom, the TIC type was efflux, efflux, efflux, and influx, respectively. [file 12880_2021_688_MOESM1_ESM.tif]
